# Differential involvement of dopamine receptor subtypes in the acquisition of Pavlovian sign-tracking and goal-tracking responses

**DOI:** 10.1007/s00213-019-5169-8

**Published:** 2019-01-25

**Authors:** Stephanie Roughley, Simon Killcross

**Affiliations:** School of Psychology, UNSW Sydney, Sydney, NSW 2052 Australia

**Keywords:** Dopamine, Sign-tracking, Pavlovian conditioning, D1, D2, Incentive salience, Learning

## Abstract

**Rationale:**

Previous work has identified that different forms of Pavlovian conditioned approach, sign-tracking and goal-tracking, are governed by distinct neurochemical mechanisms when compared in animals predisposed to learning one form vs. the other.

**Objectives:**

The present study aimed to investigate whether these are also neurochemically distinct processes in a population of animals capable of developing either response when this is manipulated via the use of distinct conditioned stimuli (CS).

**Methods:**

Rats were trained on one of two Pavlovian conditioning procedures in which the CS was either a lever, which elicits sign-tracking, or an auditory click, which elicits goal-tracking. The differential involvement of dopamine D1- and D2-receptors (D1R; D2R) in the acquisition of approach types was investigated via systemic administration of antagonists selective to one or both receptor subtypes during Pavlovian training.

**Results:**

Results indicate that dopaminergic signalling is important for the acquisition of both sign-tracking and goal-tracking responses. However, whilst development of sign-tracking to a lever depends on activity at both D1R and D2R, development of goal-tracking in response to a click was shown to depend only on activity at D1R.

**Conclusions:**

We suggest that the importance of D1R activity in both sign- and goal-tracking acquisition reflects a general role in learning Pavlovian associations, which aligns with data implicating dopamine in prediction error processes. In contrast, the selective involvement of D2R activity in sign-tracking acquisition may reflect its importance in motivational processes such as incentive salience attribution.

**Electronic supplementary material:**

The online version of this article (10.1007/s00213-019-5169-8) contains supplementary material, which is available to authorized users.

Investigations into neurochemical mechanisms underlying basic reward-learning processes provide important insights into how normal function becomes dysregulated in psychological disorders involving the reward system, such as addiction. One recent focus in this regard has been Pavlovian conditioned approach, particularly with respect to differences underpinning variation in the topography of such approach.

In Pavlovian conditioning, a conditioned stimulus (CS) develops the capacity to elicit responding on the basis of its predictive relationship with a motivationally relevant unconditioned stimulus (US). In appetitive procedures, one form of conditioned response (CR) that develops is approach behaviour during CS presentations. There is natural variation in the nature of this Pavlovian conditioned approach, in that it may be directed towards either the CS itself (sign-tracking) or the predicted location of US delivery (goal-tracking; Boakes 1977, 1979). At present, there is limited empirical evidence regarding the underlying associative structures and/or motivational processes that differentiate the two behaviours. A recent proposition, however, that the critical distinction between sign- and goal-tracking may lie in differential attribution of “incentive salience” to the CS, highlights that a greater understanding of the mechanisms underpinning this variation in responding is of particular relevance to models of addiction.

Incentive salience describes the motivational properties of a stimulus that make it attractive and “wanted”—a desire reflected in the capacity of the stimulus to motivate approach towards it, and in the exertion of effort to obtain it (Berridge 2007; Meyer et al. 2012). Whilst primary rewards (food, sex, etc.) are naturally high in incentive salience, under some circumstances this also extends to predictors of those rewards. Sign-tracking may be a useful behavioural index of situations in which a CS has been imbued with incentive salience. This is relevant to addiction, as incentive salience attributed to cues associated with abused drugs may afford such stimuli a degree of control over attention and behaviour beyond that of ordinary CSs, and behaviour motivated by the incentive salience of drug-related stimuli may be a significant contributing factor in the maintenance and relapse of drug taking (Huys et al. 2014).

Recent research highlights the importance of individual difference variation in determining an animal’s basic propensity towards acquisition of sign-tracking responses, and identifies that different neurochemical processes are required for learning in populations of animals that do and do not have this propensity. Specifically, Flagel et al. (2011) indicate that in rats predisposed to developing sign-tracking responses, dopamine is critical for the development and expression of these responses. In contrast, for rats predisposed towards goal-tracking (i.e. those that acquire goal-tracking to a cue that demonstrably supports sign-tracking), they propose that dopamine is required only for the expression of these responses, and not their development. These findings complement existing work arguing that dopamine’s function in Pavlovian reward-learning is selectively in the attribution of incentive salience (Berridge 2007), and together these findings have resulted in a novel model of addiction vulnerability in which predisposition towards dopamine-dependent attribution of incentive salience to CSs is key (Flagel et al. 2010, 2014). Furthermore, under the assumption that the attribution of incentive salience to a CS can be reliably indexed by the development of sign-tracking over goal-tracking behaviour, this has become a favoured paradigm with which to further explore neurochemical processes involved in addiction, and incentive salience more generally.

It is important to note that the logic underpinning this line of research holds specifically in circumstances where innate preferences of animals to sign- or goal-track are being exploited in order to compare these behaviours. However, there are also other factors that can impact the development of sign- vs. goal-tracking, including specific properties of the cues and/or outcomes about which an organism is learning. For example, sign-tracking behaviour is more likely to develop to manipulable, localisable cues (e.g. a lever; Holland 1980; Meyer et al. 2014), whilst goal-tracking has been shown to develop to diffuse cues (Beckmann and Chow 2015; Chow et al. 2016) and is more likely to develop to outcomes animals find more palatable (Patitucci et al. 2016). There is an open question as to how these factors interact with innate propensities in generating sign- and goal-tracking, which has important implications for how we define these behaviours and, consequently, how we study them.

Accepting the incentive salience description of sign-tracking (or at least the notion that sometimes Pavlovian reward-learning engages a motivational process over and above learning that a cue predicts reward, and which results in approach towards that cue), the fact that this can be influenced by both inherent and environmental factors raises the possibility that whilst sign-tracking behaviour may well be a good indication of a CS possessing incentive salience, goal-tracking may not necessarily be a reliable indicator of its absence. Specifically, the question arises as to whether sign-tracking requires both an inherent predisposition towards incentive salience attribution *and* the presence of a cue that supports CS-approach behaviour, or whether an inherent predisposition towards incentive salience attribution means that such animals engage a “sign-tracking” system even when responding to cues that naturally support goal-tracking behaviours (such as auditory cues).

If an animal’s predisposition alone dictates the neural system engaged for learning (e.g. to ascribe incentive salience to all CSs, or not at all), overt sign-tracking behaviour may only be observed for cues that support it, but cues that elicit US-approach (nominally “goal-tracking”) behaviour will nonetheless be controlled by one system or the other, depending on the nature of the animal. Alternatively, if CS- and US-approach behaviours themselves depend on different neural systems, it could be that the nature of the cue (rather than solely the animal’s predisposition) strongly dictates the system engaged in a particular learning situation and so even animals predisposed to sign-tracking engage goal-tracking systems in the presence of cues that favour goal-tracking behaviours.

The possibility that US-approach behaviour in sign-tracking animals might be better defined as “sign-tracking to the goal” may help explain findings indicating the involvement of dopamine in Pavlovian acquisition where the conditioned response measured was approach to the food magazine (Andrzejewski and Ryals 2016; Darvas et al. 2014; Eyny and Horvitz 2003), which would otherwise contradict the notion that dopamine is not necessary for acquisition of goal-tracking (Flagel et al. 2011). In addition, it is important to explore this question more directly for the purposes of research that seeks to increase our understanding of associative learning processes in general, as well as that which makes use of the sign-tracking/goal-tracking paradigm for modelling aspects of emotional and motivational disorders like addiction.

Some studies have utilised different CSs to promote development of sign-tracking vs. goal-tracking and do suggest that these behaviours are governed by distinct associative and motivational processes when manipulated in this fashion, within individuals (Beckmann and Chow 2015; Meyer et al. 2014). However, the neural mechanisms that underpin the development of these behaviours in such circumstances have not been extensively explored.

The current study ascertains whether selectivity in dopamine’s involvement in sign- and goal-tracking is observed when these behaviours are manipulated by the use of different CSs in a population of sign-tracking predisposed rats. If the behaviours generated by this preparation are akin to those observed in selective sign- and goal-tracking populations (Flagel et al. 2011), then dopamine should be required for the acquisition (learning) of CS-approach during a sign-tracking cue, but not for US-approach during a goal-tracking cue. However, if US-approach in sign-tracking animals is more akin to their CS-approach, then both would be expected to be dopamine-dependent. This was investigated in a series of experiments in which rats were trained to associate food reward with either a retractable lever (sign-tracking CS) or an auditory cue (goal-tracking CS), and the impact of dopamine antagonists on developing CRs was assessed.

## Methods

### Subjects

A total of 112 experimentally naïve male Wistar rats (BRC Laboratory Animal Service, University of Adelaide, SA, Australia), aged 12–16 weeks, were used. Based on extensive experience in the lab, we know that > 95% of these rats reliably develop robust sign-tracking responses to a lever CS (see Online Resource 1). Rats were housed in groups of four, in a temperature- and humidity-controlled environment (22 °C) operating on a 12-h light/dark cycle (lights on at 0700 h). Experimental procedures took place during the light cycle. Before behavioural training, rats received restricted food such that their weights were reduced to no less than 85% of free-feeding values. Water was available in home cages ad libitum. Animal procedures were carried out in accordance with the National Institute of Health Guide for the Care and Use of Laboratory Animals (NIH publications No. 80-23, revised 1996), approved by the UNSW Animal Care and Ethics Committee.

### Apparatus

Behavioural apparatus comprised eight standard operant chambers (30 cm × 24 cm × 22 cm; MED Associates Inc., St. Albans, VT), individually housed in light- and sound-attenuating compartments. Chambers were equipped with a recessed food magazine located at the bottom centre of the right hand wall, into which reward pellets could be delivered from a pellet dispenser. Head entries were detected by breaks of an infrared beam across the opening of the magazine. The auditory stimulus used for the goal-tracking procedure was a train of clicks (10 Hz) generated by the operation of a heavy-duty relay located externally to the chamber, in the rear right corner of the compartment. For sign-tracking, the CS was a retractable, stainless steel lever, located to the left of the magazine. Any contact sufficient to depress the lever was recorded as a lever-press, though such contact had no programmed consequences. Each chamber was also illuminated by a diffuse 4.2 W-house-light located at the top-centre of the left-hand wall, and was fitted with a ventilation fan that also served to mask extraneous noise. Experimental events were controlled and recorded via a PC running Med-PC software.

### Drugs

Antagonists were dissolved in 0.9% saline and injected subcutaneously 15 min prior to behavioural sessions. Doses were determined on the basis of pilot data, ensuring animals would be able to maintain adequate motor performance and be sufficiently aroused and motivated to consume food rewards. In experiments 1–3, respectively, antagonists used were α-flupenthixol (flupenthixol dihydrochloride; Sapphire Bioscience; Redfern, Australia), a non-selective dopamine receptor antagonist, administered at a dose of 0.5 mg/kg; SCH39166 (Tocris Bioscience; Bristol, UK), a selective D1R antagonist, administered at a dose of 0.0225 mg/kg; and eticlopride hydrochloride (Sigma-Aldrich; Sydney, Australia), a selective D2R antagonist, administered at a dose of 0.0125 mg/kg. Although, in the interest of simplicity, we refer here to D1- and D2-receptors, it should be noted that due to the considerable similarity between D1- and D5-receptors, and D2- and D3/D4-receptors (and consequent limitations in the specificity of dopaminergic agents), these terms relate more broadly to the D1-like and D2-like receptor families.

### Behavioural procedures

#### Pretraining

Rats were handled daily in the week preceding the onset of Pavlovian conditioning to minimise the impact of training and injection protocols (though they remained naïve to both until training began). In addition, rats were familiarised with food rewards (45 mg grain pellets; Bio-Serve, Frenchtown, NJ) for 3 days prior to Pavlovian conditioning. On the third day, this comprised a single 30-min session in the operant chambers, during which a food pellet was delivered to the food magazine approximately once every 60 s according to a variable time schedule. Following this session, all animals reliably retrieved pellets from the magazine.

#### Pavlovian conditioning

Rats were trained for 12 sessions on one of two Pavlovian conditioning procedures, differentiated by CS identity. For half the animals, the CS was a lever, for the other half, a train of clicks. There were 28 CS-US pairings per session, each consisting of a 10-s presentation of the CS that co-terminated with delivery of a single grain pellet (US). Trials were separated by a VT60 inter-trial interval (ITI; actual ITI ranged between 30 and 90 s). Animals trained with the lever CS develop a predominant lever-press CR (sign-tracking condition; see Online Resource 1), whilst those trained with the click CS develop a predominant magazine entry CR (goal-tracking condition). In each condition, animals were randomly assigned to receive injections of either saline or dopamine antagonist, administered prior to each of the first 7 sessions of training. To subsequently assess the impact this treatment had on acquisition, animals did not receive injections on sessions 8–12. In this way, behaviour could be measured in a drug-free state, such that any effects the antagonists may have on the performance of conditioned responding would not confound assessment of what the animals had learned in the preceding training sessions.

#### Data analysis

The rate (per min) of conditioned responding during the CS period was recorded for all experiments, measured as lever-pressing (sign-tracking) during lever CS presentations, and magazine entry (goal-tracking) during click CS presentations. Goal-tracking behaviour during lever CS presentations was minimal (see Online Resource 1). Measures of baseline magazine entry behaviour were recorded, taken as the rate of magazine entry responding during the 10-s period prior to CS presentation (PreCS period). Data were analysed using mixed-design, repeated measures analysis of variance (ANOVA). All relevant effects are reported in text; Online Resource 1 provides a full account of all statistics. Where relevant, significant interactions were followed-up with simple effects analysis and/or pairwise comparisons. Wherever pairwise comparisons were used, a Sidak correction for multiple comparisons was employed.

## Results

### Experiment 1: α-Flupenthixol

Experiment 1 assessed the impact of the non-selective dopamine antagonist α-flupenthixol on the acquisition of sign- and goal-tracking behaviour.

**Fig. 1:**
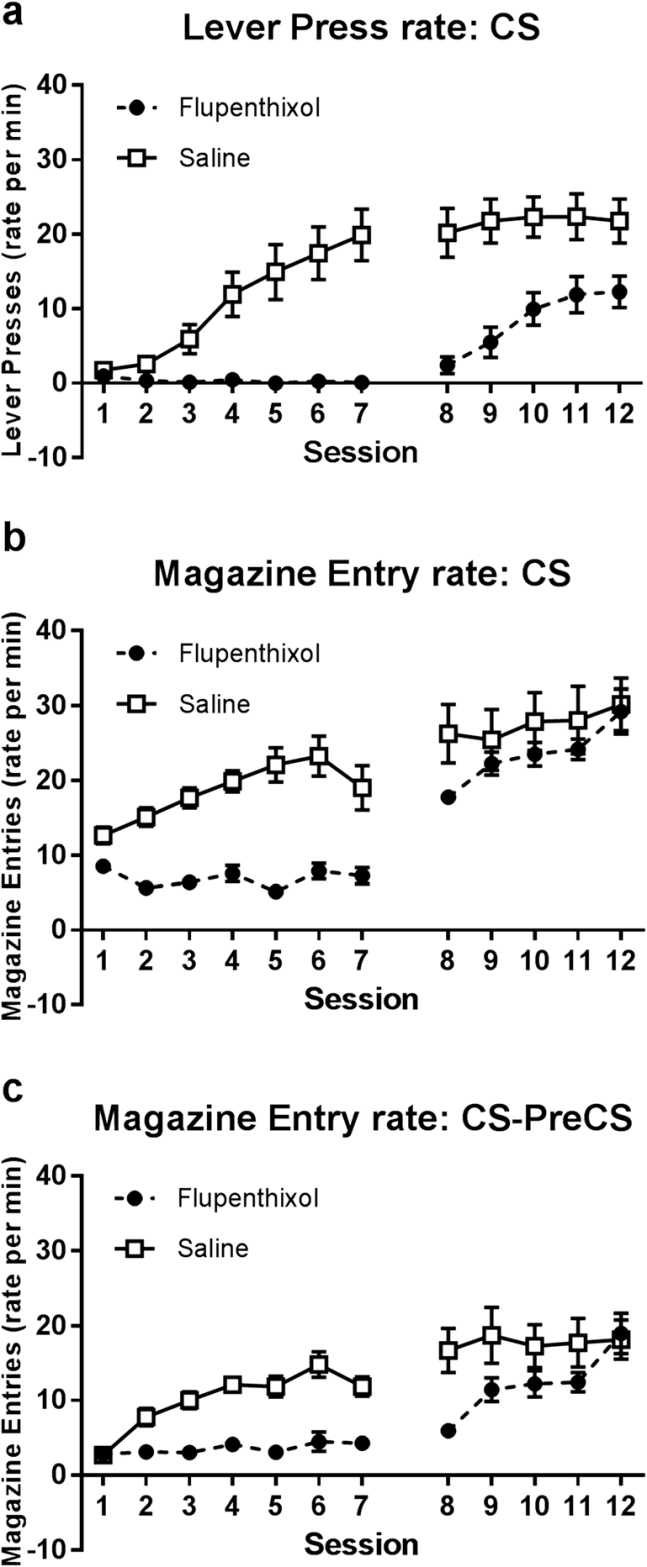
Rates of lever-pressing (sign-tracking) in the CS period for groups trained with the sign-tracking cue (panel **a**), and rates of magazine entry (goal-tracking) in the CS period (panel **b**) and the CS-PreCS period (panel **c**) for groups trained with the goal-tracking cue (*N* = 48; *n* = 12). Administration of α-flupenthixol (sessions 1–7) impaired rates of both lever-pressing and magazine entry. This impairment remained evident in the subsequent drug-free sessions (8–12) for sign-tracking, as well as goal-tracking when a CS-PreCS measure was used. Error bars represent ±SEM

Figure 1a shows the average rate of lever-press CRs across training for groups trained on the sign-tracking procedure (lever CS). Acquisition of sign-tracking was significantly impaired by α-flupenthixol treatment. Separate two-way ANOVAs performed for drug-treatment and post-treatment periods (sessions 1–7 and 8–12, respectively; between-subjects factor Group, and within-subjects factor Session) demonstrate that animals in the α-flupenthixol-treated group responded at a lower rate relative to saline-treated controls across drug-treatment sessions (main effect Group, *F*_1,22_ = 23.502, *p* < 0.001), and that this impairment remained evident in subsequent drug-free sessions (main effect Group, *F*_1,22_ = 16.120, *p* < 0.05). That the deficit was maintained on these later sessions indicates an impact of α-flupenthixol on the acquisition of sign-tracking behaviour. If the treatment affected only the performance of the sign-tracking response, and not learning, one would expect there to be no deficit in responding once drug-treatment was ceased.

Figure 1b shows the average rate of magazine entry CRs across training for groups trained on the goal-tracking procedure (click CS). Using this measure (responding during the CS period), α-flupenthixol treatment appears to impair the expression, but not acquisition, of goal-tracking CRs. Statistical analyses comprising two-way ANOVAs of drug-treatment and post-treatment periods confirm that rates of goal-tracking were significantly lower in the α-flupenthixol-treated group compared to the saline-treated group during drug-treatment sessions (main effect of Group, *F*_1,22_ = 52.532, *p* < 0.001), but *not* during the post-treatment sessions (main effect Group, *F*_1,22_ = 1.106, *p* > 0.05). However, before drawing conclusions regarding dopamine-independent acquisition of goal-tracking, it is important to consider baseline rates of responding.

The above analysis of CS responding was initially chosen in order to replicate previous work (Flagel et al. 2011) and therefore facilitate comparison between studies. However, observation of the data also indicated that α-flupenthixol treatment substantially impaired baseline levels of magazine approach behaviour (see Online Resource 1). This is an issue as by definition, acquisition of a CR should involve a selective increase in the target behaviour during the period of CS presentation—CRs should be greater during the CS than at other points in time, reflecting sensitivity to the relationship of CS and reward. As is common practice in appetitive learning literature, we addressed this by subtracting a measure of baseline performance from the observed change in CS responding (Fig. 1c).

Figure 1c shows the average rate of magazine entry during CS presentations after subtracting baseline response rates across matched PreCS periods. Having accounted for baseline responding, clear deficits in goal-tracking acquisition are revealed following α-flupenthixol treatment. Two-way ANOVAs performed for drug-treatment and post-treatment periods show that rates of goal-tracking were significantly lower in the α-flupenthixol-treated group compared to the saline-treated group during drug-treatment sessions (main effect Group, *F*_1,22_ = 45.834, *p* < 0.001). In post-treatment sessions, a significant Group by Session interaction was observed (*F*_4,88_ = 4.541, *p* < 0.05), as well as a main effect of Session (*F*_4,88_ = 6.740, *p* < 0.001), though not of Group (*F*_1,22_ = 3.166, *p* = 0.089). Simple effects analysis clarifies that the effect of Session is selective to the α-flupenthixol-treated group (*F*_4,19_ = 10.329, *p* < 0.001; *F* < 1 for saline), and that on the first post-treatment session (8), responding was significantly lower in the α-flupenthixol compared to the saline-treated group (*F*_1,22_ = 12.466, *p* < 0.05). Animals treated with α-flupenthixol during training acquired goal-tracking CRs across the post-treatment period, but started at a deficit relative to control animals.

It should be noted here that in the context of the present design, this same analysis cannot be achieved for sign-tracking, as the lever manipulandum is not present during periods other than CS presentation. However, this is also less critical; as the data stands, there was no observed increase from drug-treatment to post-treatment sessions that might reflect intact acquisition of sign-tracking responses. Should α-flupenthixol impair baseline (unconditioned) tendencies to lever-press, this would simply serve to enhance the deficit in acquisition that was observed. Overall, therefore, the present data indicate that dopamine antagonism via α-flupenthixol treatment impairs acquisition of both sign-tracking and goal-tracking CRs.

### Experiment 2: SCH39166

Experiments 2 and 3 assessed whether the finding of experiment 1—that dopamine is important in the acquisition of both sign- and goal-tracking responses—could be isolated to activity at dopamine receptor subtypes. Experiment 2 replicated experiment 1, but experimental groups were treated with the dopamine D1R antagonist SCH39166.

**Fig. 2:**
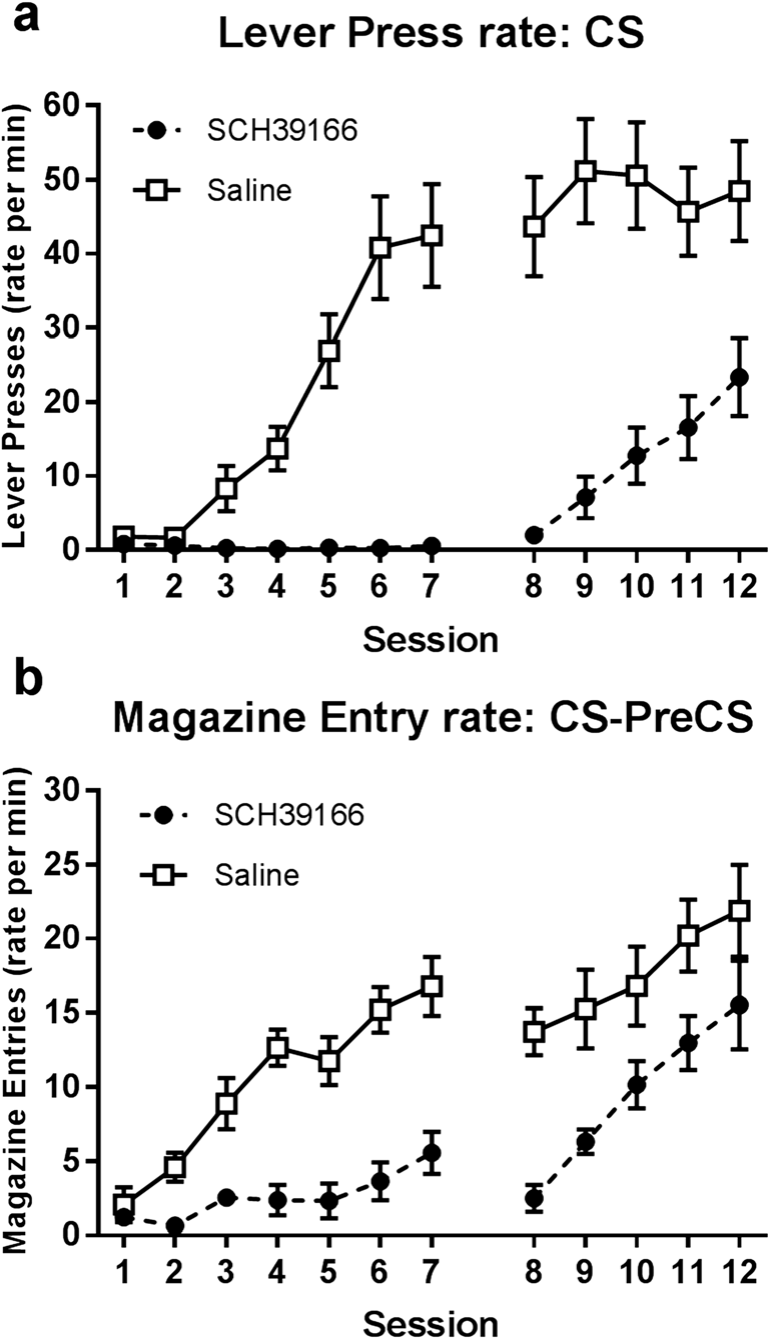
Panel **a** displays rates of lever-pressing during the CS for groups trained on the sign-tracking cue, whilst panel **b** displays rates of magazine entry (CS-PreCS measure) for groups trained on the goal-tracking cue (*N* = 32; *n* = 8). Administration of SCH39166 (sessions 1–7) impaired rates of both lever-pressing and magazine entry, and this impairment remained evident in both cases during subsequent drug-free sessions (8–12). Error bars represent ±SEM

Figure 2 displays the average rates, across training, of lever-press CRs for groups in the sign-tracking condition (Fig. 2a; lever CS) and magazine entry CRs for groups in the goal-tracking condition (Fig. 2b; CS-PreCS measure; click CS). Administration of SCH39166 impaired the acquisition of both sign-tracking and goal-tracking responses. Separate two-way ANOVAs performed for drug-treatment and post-treatment periods (sessions 1–7 and 8–12, respectively; between-subjects factor Group, and within-subjects factor Session) indicate that SCH39166-treated animals responded at a lower rate relative to saline-treated controls during the drug-treatment period in both sign-tracking (main effect Group, *F*_1,14_ = 32.572, *p* < 0.001) and goal-tracking conditions (main effect Group, *F*_1,14_ = 48.876, *p* < 0.001). Furthermore, this impairment remained evident in the post-treatment period for both sign-tracking (main effect Group, *F*_1,14_ = 25.134, *p* < 0.001) and goal-tracking conditions (main effect Group, *F*_1,14_ = 10.989, *p* < 0.05).

### Experiment 3: Eticlopride

**Fig. 3:**
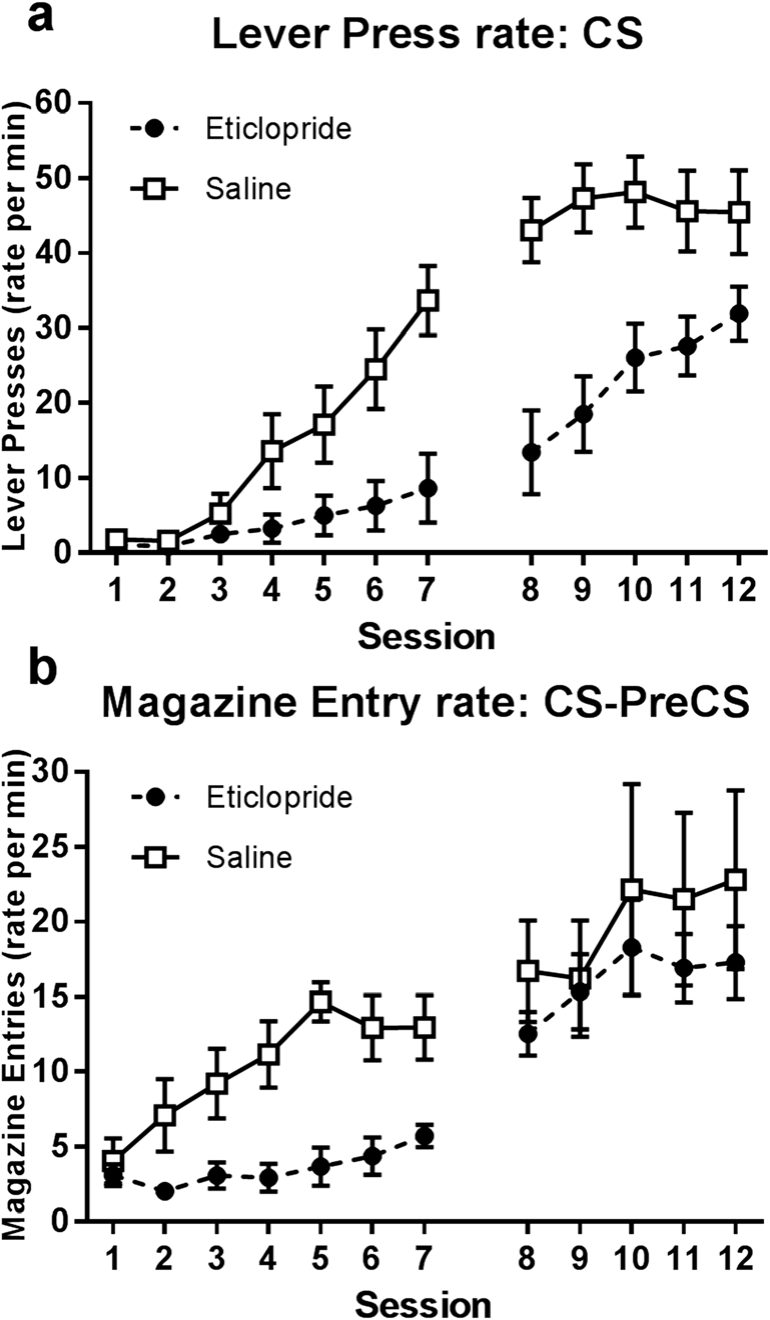
Panel **a** displays rates of lever-pressing during the CS for groups trained on the sign-tracking cue, whilst panel **b** displays rates of magazine entry (CS-PreCS measure) for groups trained on the goal-tracking cue (*N* = 32; *n* = 8). Administration of eticlopride (sessions 1–7) impaired rates of both lever-pressing and magazine entry. This impairment remained evident during subsequent drug-free sessions (8–12) in sign-tracking animals, but not goal-tracking animals. Error bars represent ±SEM

Experiment 3 assessed the impact of the dopamine D2R antagonist eticlopride on the acquisition of sign- and goal-tracking CRs. Figure 3a shows the average rate of lever-press CRs for groups in the sign-tracking condition (lever CS). Acquisition of sign-tracking was significantly impaired by eticlopride treatment. Separate two-way ANOVAs performed for drug-treatment and post-treatment periods (sessions 1–7 and 8–12, respectively; between-subjects factor Group, and within-subjects factor Session) indicate that eticlopride-treated animals responded at a lower rate relative to saline-treated controls during the drug-treatment period (main effect Group, *F*_1,14_ = 7.609, *p* < 0.05), and the subsequent post-treatment sessions (main effect Group, *F*_1,14_ = 13.455, *p* < 0.05).

Figure 3b shows the average rate of magazine entry CRs (CS-PreCS measure) across training for groups in the goal-tracking condition (click CS). Treatment with eticlopride impaired the expression, *but not* the acquisition, of goal-tracking responses. Statistical analyses comprising two-way ANOVAs of drug-treatment and post-treatment periods show that rates of goal-tracking responding were significantly lower in the eticlopride-treated group compared to the saline-treated group during drug-treatment sessions (main effect of Group, *F*_1,14_ = 14.205, *p* < 0.05). However, and in contrast to the previous two experiments, this was not observed during the post-treatment sessions; here, there was no longer any significant difference in responding between groups (main effect Group, *F* < 1). There was no evidence of a deficit in goal-tracking CRs once eticlopride was no longer being administered, suggesting that the impairment observed during drug-treatment sessions was a performance deficit only.

## Discussion

The results of this study indicate that dopamine is important in the acquisition of CRs elicited by both sign-tracking (lever) and goal-tracking (click) CSs. More specifically, whilst both D1R and D2R activity was shown to be important for acquisition of CS-approach during presentation of a sign-tracking cue, only D1R activity was necessary for the acquisition of US-approach during presentation of a goal-tracking cue. D2R antagonism reduced the performance of goal-tracking CRs, but there was no evidence that acquisition was impaired.

These findings are broadly in line with other work that has explored the neurochemical mechanisms underlying sign- and goal-tracking behaviour (Danna and Elmer 2010; Flagel et al. 2011; Holden and Peoples 2010; Lopez et al. 2015), supporting the conclusion that these processes are differentially modulated by dopamine—with sign-tracking being more susceptible to disruption by dopaminergic manipulation than is goal-tracking. However, our results do suggest more specificity than indicated previously. In particular, Flagel et al. (2011) demonstrated that dopamine transmission was necessary for the acquisition of sign-tracking, but not at all for goal-tracking. In the present study, this dissociation was observed only at the level of dopamine D2R; the results of experiments 1 and 2 clearly indicate a role for dopamine generally, and D1R specifically, in the acquisition of goal-tracking *and* sign-tracking behaviour.

As raised earlier, one potential explanation for the difference in these findings is that because animals used in these studies would all preferentially develop sign-tracking in the presence of cues that support sign-tracking, it is possible that the US-approach behaviour elicited in response to auditory cues in this study may actually be another form of sign-tracking. This relates to the proposal that the distinction between the two behaviours lies in incentive salience attribution; in the absence of a discrete, manipulable CS to be a target for incentive salience, it is possible that predisposed animals attribute incentive salience to the US location and, in essence, “sign-track” to the goal.

In the context of the present findings, however, this argument is relatively weak. The dissociation in dopaminergic control over the CS- and US-approach behaviours shown in this study clearly indicates that these behaviours, however characterised, *are* driven by different underlying processes. This is also supported by previous work showing both behavioural and neurochemical dissociations between sign-tracking and goal-tracking behaviours when these are manipulated in a manner similar to this study (Beckmann and Chow 2015; Chow et al. 2016; Meyer et al. 2014).

Hence, present and previous findings indicate that differences in neurochemical control over US-approach in animals predisposed to sign-tracking or goal-tracking cannot be accounted for by the explanation that CS- and US-approach behaviours are actually both underpinned by a common, dopamine-dependent, process (namely incentive salience attribution) in animals predisposed to sign-tracking. In animals predisposed to sign-tracking, D2R antagonism blocked acquisition of sign-tracking but not goal-tracking behaviour. If CS- and US-approach in this study are both forms of “sign-tracking”, in the sense that both are due to acquisition of cue-related incentive salience, this would then require another explanation for the differences in dopaminergic control over the acquisition of these different “sign-tracking” CRs. For example, sign-tracking to a cue more distal to reward (e.g. a lever) may be fundamentally different to sign-tracking to a cue more proximal to reward (e.g. the food magazine).

An alternative reason for differences in present findings compared to those of Flagel et al. (2011) is that deficits in goal-tracking acquisition under dopamine antagonism were revealed only after accounting for changes in rates of baseline magazine approach. When responding during the CS period alone was analysed, our results match previous findings, but to do so ignores critical differences in baseline responding. It could also be that the difference in dopaminergic control over learning in sign-tracker vs. goal-tracker *animals* is independent of the behavioural processes that are being measured. The selective populations of sign- and goal-tracking rats used in previous studies were bred on the basis of their responsivity to novelty; there may be something in the nature of low-responsive animals that means dopamine activity is not required to learn Pavlovian associations, but this is not related to the mechanism responsible for generating CS- compared to US-approach. Either explanation would accommodate the findings of Flagel et al. (2011) whilst also allowing one to retain the notion that sign- and goal-tracking behaviours—operationalised simply based on observed topography of behaviour—are a consequence of different underlying processes being engaged during learning.

Conserving the behavioural distinction drawn between sign- and goal-tracking would maintain that in this study, CS- and US-approach behaviours both depend upon learning a predictive association between a CS and appetitive US, but the development of CS-approach also involves some additional motivational process during acquisition that renders the CS attractive. A parsimonious interpretation of the present data within this framework would therefore be that in the context of acquisition, D1R are critical for forming an association, whilst D2R are more selectively involved in incentive motivational components of learning. This does not deny the importance of animals predisposed to sign-tracking or goal-tracking; the former population is predisposed to engage learning processes that assign incentive salience to cues, whilst the latter is not. In addition, this argument is particularly appealing in terms of its power to accommodate findings from a range of other work focussed around the role of dopamine in learning and behaviour.

The interpretation just outlined accords with previous studies that have demonstrated the general importance of dopamine during the acquisition of conditioned approach, irrespective of whether this approach was directed towards the CS (sign-tracking; Dalley et al. 2002; Di Ciano et al. 2001; Parkinson et al. 2002) or US (goal-tracking; Darvas et al. 2014). Other studies have also investigated the involvement of particular dopamine receptor subtypes in these behaviours, and collectively support the finding that D1R activity is important for acquisition of goal-tracking (Andrzejewski and Ryals 2016) as well as sign-tracking responses (Clark et al. 2013; Dalley et al. 2005), whereas the involvement of D2R appears more selective (Banasikowski et al. 2010; Beninger and Hahn 1983; Eyny and Horvitz 2003). By way of contrast, these same findings are problematic for the notion that dopamine is only necessary for sign-tracking, not goal-tracking, unless one appeals to the argument that goal-tracking in these instances may be “sign-tracking to the goal”, a notion which cannot accommodate the present findings.

Our results also extend work proposing a role for phasic dopamine release in formation of Pavlovian associations. Associative models suggest that acquisition of a CS-US association progresses by a process of error correction, whereby the discrepancy between the expected and experienced outcome following a particular stimulus or event (i.e. the prediction error) updates expectancy to reduce that discrepancy on the next encounter. It has long been observed that changes in phasic dopamine release following reward (US) and signal (CS) presentation correlate with calculated changes in prediction error and expectancy across learning (Schultz 2007; Schultz and Dickinson 2000), and recent evidence strongly suggests this relationship is causal (Chang et al. 2016; Steinberg et al. 2013). If phasic signalling by dopamine neurons is critical for learning CS-US relationships, then this would be relevant to acquisition of both sign-tracking and goal-tracking. Further, given that stimulation of D1R is selectively achieved via the heightened concentrations of extracellular dopamine that accompanies phasic burst firing of dopamine neurons (Dreyer et al. 2010), D1R activity is likely to be particularly important.

Proponents of the incentive salience hypothesis of dopamine function put forward that these patterns of dopamine signalling reflect the process of incentive salience attribution to a CS, rather than acquisition of a CS-US association. Some support for this notion is provided by Flagel et al. (2011, and similar findings from Singer et al. 2016), in which dopamine release in the nucleus accumbens core was observed to transition from US to CS across conditioning for sign-tracking animals but not goal-tracking animals (in which dopamine release remained evident following both CS and US presentations even at the end of conditioning). This is corroborated by other literature implicating the accumbens core in incentive motivational processes (Aitken et al. 2016; Cardinal et al. 2002; Collins et al. 2016; Fraser and Janak 2017), although it does not rule out learning-related functions of dopaminergic prediction error signals elsewhere in the brain (Tian et al. 2016). Furthermore, Singer et al. (2016) found that patterns of dopamine release in sign-trackers changed to resemble the pattern previously observed for goal-trackers when the lever CS was covered such that animals could still approach the cue, but could not bite or manipulate it. This raises two possibilities. First, if covering the lever reduced the predictive properties of the CS and so degraded the strength of the conditioned association, then the “goal-tracker” pattern perhaps simply reflected a reduced strength of basic conditioned association in those animals (although we would note that the strength of association is difficult to determine without appropriate baseline data). Second, the “sign-tracker” pattern could be related to the consummatory actions (biting, chewing, etc.) typically engaged during sign-tracking behaviour. These remain opportunities for future research.

Additional evidence supporting the importance of dopamine in prediction error mechanisms that drive associative formation—independent of a role in motivational processes—can be found in several recent studies (Sharpe et al. 2017a, b) that show dopaminergic activity is critical for neutral-valence stimulus-stimulus learning. These studies look at learning of a predictive association that does not, in and of itself, support any kind of motivated conditioned response, and as such provide an additional challenge for the proposal that dopamine’s function is selectively in the process of incentive salience attribution (and the related suggestion that instances where dopamine is implicated in US-approach acquisition can be explained as sign-tracking to the goal; Berridge 2007; Flagel et al. 2011). In this regard, whilst our demonstration of the importance of D1R activity in the acquisition of both sign-tracking and goal-tracking behaviour is perhaps not unexpected (Andrzejewski and Ryals 2016; Clark et al. 2013; Dalley et al. 2005), it similarly supports a role for dopamine in learning-related aspects of Pavlovian approach acquisition, rather than solely incentive salience processes.

The present results do not, however, deny an important role for dopamine in incentive salience mechanisms, and in this way also extend the work that implicates dopamine in these processes (Berridge 2007). If cues to which animals sign-track are presumed to possess incentive salience, whilst those to which animals goal-track do not, then the finding that D1R antagonism blocked acquisition of both behaviours, together with the finding that D2R were differentially recruited for acquisition of these behaviours, really suggests that the role of dopamine cannot be defined in terms of one function or another. Accordingly, it is unlikely that dopamine is *exclusively* involved in incentive salience attribution—though it is also unlikely that dopamine is exclusively involved in learning-related prediction error processes.

Understanding the neurobehavioural mechanisms that produce variation in Pavlovian conditioned approach behaviour may be particularly important for the study of disorders, such as addiction, that involve dysregulation of reward-learning processes. Whilst previous work has focused on individual difference factors that drive development of these behaviours, the study of parallel mechanisms that can produce these behaviours within a given individual is also valuable. The present data indicate that in animals demonstrably capable of developing either CS-approach or US-approach behaviour, the acquisition of these behaviours is differentially mediated by dopaminergic activity. Whilst activity at dopamine D1R is important for the development of both CS- and US-approach behaviour, activity at D2R is important only in the acquisition of CS-approach. We tentatively suggest this reflects a role for D1R in learning Pavlovian CS-US associations, which underpins the acquisition of CRs in general, and for D2R in a motivational process during acquisition (such as incentive salience attribution) that is critical for generating attraction towards Pavlovian CSs.

## Electronic supplementary material

ESM 1 Although all relevant data and statistics are reported in text, Online Resource 1 provides a full description of all statistical output along with associated data in accompanying figures. (PDF 280 kb)
