# Supplementary material for: Differential involvement of dopamine receptor subtypes in the acquisition of Pavlovian sign-tracking and goal-tracking responses
Source: Psychopharmacology (Berl). 2019 Jan 25;236(6):1853–62. doi: 10.1007/s00213-019-5169-8 (PMC6602989; doi:10.1007/s00213-019-5169-8)
Supplement: Supplementary file 1 — Although all relevant data and statistics are reported in text, Online Resource 1 provides a full description of all statistical output along with associated data in accompanying figures. (PDF 280 kb) [file 213_2019_5169_MOESM1_ESM.pdf]

Supplemental Material

Psychopharmacology

Differential involvement of dopamine receptor subtypes in the acquisition of Pavlovian sign-tracking and goal-tracking responses

Stephanie Roughley, PhD

School of Psychology, UNSW Sydney, Sydney, NSW 2052, Australia

Simon Killcross, PhD

School of Psychology, UNSW Sydney, Sydney, NSW 2052, Australia

E-mail: s.killcross@unsw.edu.au

Tel: +61 2 9385 3034

Fax: +61 2 9385 1193

Methods

Profile of Sign-Tracking Wistar rats

We have run a large number of experiments utilizing a sign-tracking protocol in the lab, and consistently find that nearly all of the Wistar rats from our supplier (BRC Laboratory Animal Service, University of Adelaide, SA, Australia) acquire a robust sign-tracking response that comes to dominate almost exclusively over goal-tracking responding. The typical behavioural profile is as follows: in the first few training sessions we observe an initial increase in both lever-pressing (sign-tracking) and magazine approach (goal-tracking) during CS presentation. However, whilst sign-tracking behaviour continues to increase across training, rates of goal-tracking gradually decline. By the 8th session of training, animals respond on the lever at a rate of on average 27.3 lever-presses per minute, whilst goal-tracking has dropped to on average 4.1 magazine-entries per minute. With further training, magazine-entry behaviour actually becomes suppressed during the CS period, relative to baseline rates of responding (PreCS period; on average 5.1 magazine-entries per minute on the 8th session).

These averages were calculated using data from the control animals in experiments that were conducted in the lab (currently unpublished data) using the sign-tracking paramaters outlined here (i.e. lever CS as described, presented for 10s per trial, with short ~60s ITI). These data are presented in Figure S1, which shows rates of lever-press and magazine-entry responding over 12 sessions of training (some experiments did train longer than this).

In addition to looking at the typical behavioural profile, we also calculated a response bias for each animal, based on the total number of lever-presses and magazine-entries performed during CS presentations in a given session, averaged across the final three sessions of training. This was calculated as [lever-presses – magazine-entries] / [lever-presses + magazine-entries], such that +1.0 would indicate exclusive sign-tracking, -1.0 would indicate exclusive goal-tracking, and 0 would indicate no bias. Of a total n = 251, 233 animals displayed a preference for sign-tracking (bias score > 0.3), 7 displayed a preference for goal-tracking (bias score < -0.3), and 11 displayed no preference (bias score between -0.3 and 0.3). This equates to 92.8% we would class as "sign-trackers", 2.8% "goal-trackers" and 4.4% indeterminate. It is worth noting that of the animals that displayed no strong preference either way, all nonetheless demonstrated robust lever-press behaviour. Based on these data, we reasonably expect that in approximately 97% of cases an animal will develop a substantial sign-tracking response that can be measured.

Results

Experiment 1

Sign-Tracking: Additional statistics on lever-press responding

For lever-press responding during the CS in groups trained on the sign-tracking procedure, there were also main effects of Session in both the drug-treatment and post-treatment periods ($F_{6,132} = 14.718, p < 0.001$; $F_{4,88} = 8.969, p < 0.001$, respectively) in addition to the main effects of Group. Furthermore, simple effects analyses of significant Session by Group interactions ($F_{6,132} = 16.466$, $p < 0.001$; $F_{4,88} = 4.613$, $p < 0.05$, respectively) revealed that an increase in responding across session was specific to the saline-treated group during the treatment period ($F_{6,17} = 10.215, p < 0.001$; $F < 1$ for α-flupenthixol) and the α-flupenthixol-treated group during post-treatment period ($F_{4,19} = 7.275, p < 0.05$; $F < 1$ for saline).

Sign-Tracking: Magazine-entry responding

For magazine entry responding during lever CS presentations in groups trained on the sign-tracking procedure (Figure S2), two-way ANOVAs performed for drug-treatment and post-treatment periods (sessions 1-7 and 8-12, respectively) indicate that α-flupenthixol-treated animals responded at a lower rate relative to saline-treated controls during the drug-treatment period ($F_{1,22} = 7.975$, $p < 0.05$) and at a higher rate during the post-treatment period ($F_{1,22} = 88.494, p < 0.001$). Across the drug-treatment sessions, there was also a significant main effect of Session ($F_{6,132} = 3.720, p < 0.05$), reflecting a decline in magazine entry responding (linear trend $F_{1,22} = 7.709, p < 0.05$), and a significant Session by Group interaction ($F_{6,132} = 6.139, p < 0.001$). Simple effects analysis demonstrates that the effect of Session was significant in both saline- and α-flupenthixol-treated groups ($F_{6,17} = 8.626, p < 0.001$ and $F_{6,17} = 5.232, p < 0.05$, respectively). However, differences in the change across sessions for saline- vs. α-flupenthixol-treated groups are indicated by pairwise comparisons that show responding for the saline-treated group was higher than in the α-flupenthixol-treated group on sessions 1, 2, 3, and 5 (minimum $F_{1,22} = 5.332, p < 0.05$), but not on sessions 4, 6, and 7 (maximum $F_{1,22} = 1.920, p > 0.05$). In the post-treatment sessions, the main effect of Session was again significant ($F_{4,88} = 13.090$, $p < 0.001$), reflecting further decline in magazine entry responding (linear trend $F_{1,22} = 22.332, p < 0.001$), but there was no significant interaction ($F_{4,88} = 2.357, p > 0.05$).

Goal-Tracking: Additional statistics on magazine-entry responding

For magazine entry responding during the CS period for animals trained on the goal-tracking procedure, a main effect of Session was also observed in both drug-treatment and post-treatment periods ($F_{6,132} = 5.358$, $p < 0.001$; $F_{4,88} = 7.259$, $p < 0.001$, respectively). There was also a Session by Group interaction in the treatment period ($F_{6,132} = 6.661$, $p < 0.001$; $F_{4,88} = 1.679$, $p > 0.05$ in the post-treatment period), but follow-up simple effects did not reveal any meaningful differences.

For magazine entry responding during the PreCS period for groups trained on the goal-tracking procedure (Figure S3), two-way ANOVAs performed for drug-treatment and post-treatment periods (sessions 1-7 and 8-12, respectively) revealed a significant main effect of Group ($F_{1,22} = 36.148$, $p < 0.001$) in the drug-treatment period, whereby responding was lower in the α-flupenthixol-treated group compared to the saline-treated group. There was also a main effect of Session ($F_{6,132} = 3.231$, $p < 0.05$), but no Session by Group interaction ($F_{6,132} = 1.755$, $p > 0.05$). In the post-treatment period, no effects were significant (Session, $F_{4,88} = 1.710$, $p > 0.05$; Group, $F < 1$; Session by Group interaction, $F_{4,88} = 2.033$, $p > 0.05$).

Experiment 2

Sign-Tracking: Additional statistics on lever-press responding

For lever-press responding during the CS in groups trained on the sign-tracking procedure, there was a significant main effect of Session across the drug-treatment and post-treatment periods ($F_{6,84} = 28.757$, $p < 0.001$ and $F_{4,56} = 7.913$, $p < 0.001$, respectively) in addition to main effects of Group. Furthermore, there was a Session by Group interaction in both periods ($F_{6,84} = 29.254$, $p < 0.001$ and $F_{4,56} = 5.775$, $p < 0.05$, respectively), which for the drug-treatment period simple effects analysis revealed was a function of a significant effect of Session in the saline-treated group ($F_{6,9} = 8.120$, $p < 0.05$), but not the SCH39166-treated group ($F < 1$). Simple effects analysis of the interaction in the post-treatment period did not reveal any meaningful differences.

Sign-Tracking: Magazine-entry responding

For magazine entry responding during lever CS presentations in groups trained on the sign-tracking procedure (Figure S4), two-way ANOVAs performed for drug-treatment and post-treatment periods (sessions 1-7 and 8-12, respectively) indicate that SCH39166-treated animals responded at a lower rate relative to saline-treated controls during the drug-treatment period

($F_{1,14}$ = 29.405, $p < 0.001$), and at a higher rate during the post-treatment period ($F_{1,14}$ = 61.711, $p < 0.001$). Across the drug-treatment sessions, there was also a significant main effect of Session ($F_{6,84}$ = 9.071, $p < 0.001$), reflecting an initial increase, followed by decline, in magazine entry responding (quadratic trend, $F_{1,14}$ = 25.372, $p < 0.001$), and a significant Session by Group interaction ($F_{6,84}$ = 11.648, $p < 0.001$). Simple effects analysis demonstrates that the effect of Session was significant in the saline- but not the SCH39166-treated group ($F_{6,9}$ = 11.404, $p = 0.001$ and $F_{6,9}$ = 1.095, $p > 0.05$, respectively); responding in the SCH39166-treated group remained at a similarly low level throughout the drug-treatment period. In the post-treatment sessions there was also a significant main effect of Session ($F_{4,56}$ = 6.206, $p < 0.001$), again reflecting an initial increase followed by decline (quadratic trend, $F_{1,14}$ = 15.506, $p = 0.001$), as well as a Session by Group interaction ($F_{4,56}$ = 3.703, $p < 0.05$). In this instance, simple effects analysis revealed that the effect of Session was attributable to the SCH39166-treated group ($F_{4,11}$ = 17.045, $p < 0.001$), not the saline-treated group ($F < 1$); responding in the saline-treated group remained at a similarly low level throughout this period.

Goal-Tracking: Additional statistics on magazine-entry responding

For magazine entry responding during the CS-PreCS period for animals trained on the goal-tracking procedure, there was a main effect of Session across both the drug-treatment and post-treatment periods ($F_{6,84}$ = 20.117, $p < 0.001$ and $F_{4,56}$ = 15.602, $p < 0.001$, respectively). There was also a Session by Group interaction in the drug-treatment period ($F_{6,84}$ = 7.071, $p < 0.001$) but not the post-treatment period ($F < 1$). Simple effects analysis showed this interaction was due to a significant Session effect in the saline-treated group ($F_{6,9}$ = 11.335, $p < 0.05$) but not the SCH39166-treated group ($F_{6,9}$ = 2.170, $p > 0.05$).

Looking at PreCS responding only (Figure S5a), two way ANOVAs performed for the drug-treatment and post-treatment periods (session 1-7 and 8-12, respectively) indicate that magazine entry responding in the SCH39166-treated groups was significantly lower than in saline-treated groups during the drug-treatment period ($F_{1,14}$ = 26.683, $p < 0.001$), but significantly higher during the post-treatment period ($F_{1,14}$ = 13.058, $p < 0.05$). In the drug-treatment period the effect of Session was also significant ($F_{6,84}$ = 5.690, $p < 0.001$), though this did not reflect any interpretable change in the pattern of responding (significant trends at the 1st, 2nd, 3rd, and 4th order; minimum $F_{1,14}$ = 4.733, $p < 0.05$). There was no Session by Group interaction ($F < 1$). In the post-treatment period no other effects were significant (both $F < 1$).

Looking only at responding in the CS period (Figure S5b), similar analyses revealed that responding in the SCH39166-treated groups was significantly impaired relative to saline-treated controls during the drug-treatment period ($F_{1,14} = 58.089$, $p < 0.001$), but not during the post-treatment period ($F_{1,14} = 1.745$, $p > 0.05$). In the drug-treatment period, there was also a significant effect of Session ($F_{6,84} = 19.273$, $p < 0.001$), reflecting an increase in responding across training (linear trend, $F_{1,14} = 61.494$, $p < 0.001$). There was also a significant Session by Group interaction ($F_{6,84} = 5.020$, $p < 0.001$), but simple effects analysis did not reveal any meaningful group differences in the change in responding across sessions. In the post-treatment period, there was a main effect of Session ($F_{4,56} = 13.447$, $p < 0.001$), reflecting an increase in responding as training progressed (linear trend, $F_{1,14} = 22.766$, $p < 0.001$), but no Session by Group interaction ($F_{4,56} = 1.055$, $p > 0.05$), indicating that this change did not differ as a function of group.

Experiment 3

Sign-Tracking: Additional statistics on lever-press responding

For lever-press responding during the CS in groups trained on the sign-tracking procedure, the analysis reveals that in addition to main effects of Group, there were also main effects of session in both the drug-treatment and post-treatment periods ($F_{6,84} = 22.644$, $p < 0.001$; $F_{4,56} = 6.760$, $p < 0.001$, respectively). Simple effects analysis of the Session by Group interaction in each case ($F_{6,84} = 8.796$, $p < 0.001$; $F_{4,56} = 4.619$, $p < 0.05$, respectively) revealed that the increase in responding across session was specific to the saline-treated group during the treatment period ($F_{6,9} = 6.562$, $p < 0.05$; $F < 1$ for eticlopride) and the eticlopride-treated group during post-treatment period ($F_{4,11} = 4.160$, $p < 0.05$; $F_{4,11} = 1.010$, $p > 0.05$ for saline).

Sign-Tracking: Magazine-entry responding

Figure S6 displays the average rates of magazine entry responding during lever CS presentations for groups trained on the sign-tracking procedure. Two-way ANOVAs performed for drug-treatment and post-treatment periods (sessions 1-7 and 8-12, respectively) show that eticlopride-treated animals responded at a lower rate relative to saline-treated controls during the drug-treatment period ($F_{1,14} = 22.062$, $p < 0.001$) and at a higher rate during the post-treatment period ($F_{1,14} = 15.500$, $p = 0.001$). Across the drug-treatment sessions, there was also a significant main effect of Session ($F_{6,84} = 7.861$, $p < 0.001$) and a significant Session by Group

interaction ($F_{6,84} = 4.768$, $p < 0.001$). Simple effects analysis demonstrates that the effect of Session was significant in both saline- and eticlopride-treated groups ($F_{6,9} = 5.709$, $p < 0.05$ and $F_{6,9} = 3.647$, $p < 0.05$, respectively). However, differences in the change across sessions for saline- vs. eticlopride-treated groups are indicated by pairwise comparisons that show responding for the saline-treated group was higher than in the α-flupenthixol-treated group on sessions 1, 2, 3, and 4 (minimum $F_{1,14} = 7.479$, $p < 0.05$), but not on sessions 5, 6, and 7 (maximum $F_{1,14} = 3.359$, $p > 0.05$) as responding in the saline-treated group declined. In the post-treatment sessions, the main effect of Session was again significant ($F_{4,56} = 17.903$, $p < 0.001$), reflecting further decline in magazine entry responding (linear trend, $F_{1,14} = 24.349$, $p < 0.001$). There was also a significant Session by Group interaction ($F_{4,56} = 4.705$, $p < 0.05$), but simple effects analysis did not reveal any meaningful group differences in the change in responding across sessions.

Goal-Tracking: Additional statistics on magazine-entry responding

In addition to the main effect of Group, for magazine entry responding during the CS-PreCS period in groups trained on the goal-tracking procedure, there was a main effect of Session ($F_{6,84} = 9.278$, $p < 0.001$) and Session by Group interaction ($F_{6,84} = 4.461$, $p < 0.05$) in the treatment sessions. Follow-up analysis showed that the session effect was specific to an increase in the saline-treated group ($F_{6,9} = 11.693$, $p < 0.05$; $F_{6,9} = 1.891$; $p > 0.05$ for eticlopride). In the post-treatment period, there was also a main effect of Session ($F_{4,56} = 3.977$, $p < 0.05$), but no Session by Group interaction ($F < 1$).

Looking at responding during the PreCS period only (Figure S7a), two way ANOVAs performed for the drug-treatment and post-treatment periods (session 1-7 and 8-12, respectively) indicate that responding in the eticlopride-treated group was significantly lower than in saline-treated group during the drug-treatment period ($F_{1,14} = 33.057$, $p < 0.001$), but not during the post-treatment period ($F < 1$). In neither the drug-treatment period nor the post-treatment period was there any effect of Session ($F_{1,14} = 1.161$, $p > 0.05$ and $F_{1,14} = 1.834$, $p > 0.05$, respectively) or Session by Group interaction ($F_{1,14} = 1.684$, $p > 0.05$ and $F < 1$, respectively).

For responding during the CS period only (Figure S7b), analyses similarly revealed that rates of magazine entry in the eticlopride-treated group was significantly impaired relative to saline-treated controls during the drug-treatment period ($F_{1,14} = 34.211$, $p < 0.001$), but not during the post-treatment period ($F < 1$). In the drug-treatment period, there was also a significant effect

of Session ($F_{6,84} = 5.731$, $p < 0.001$), reflecting an increase in responding across training (linear trend, $F_{1,14} = 33.404$, $p < 0.001$). There was also a significant Session by Group interaction ($F_{6,84} = 4.433$, $p = 0.001$), suggesting that the change in responding across sessions differed between eticlopride- and saline-treated groups. However, simple effects analysis indicates this change was significant in both cases ($F_{6,9} = 6.561$, $p < 0.05$ and $F_{6,9} = 5.477$, $p < 0.05$, respectively). In the post-treatment period, there was a main effect of Session ($F_{4,56} = 5.522$, $p = 0.001$), reflecting an increase in responding as training progressed (linear trend, $F_{1,14} = 8.488$, $p < 0.05$), but no Session by Group interaction ($F < 1$), indicating that this change did not differ as a function of group.

Fig. S1 Rates of lever pressing (sign-tracking) and magazine entry (goal-tracking) during the CS period, in addition to baseline magazine entry behaviour during the PreCS period, for control animals in a collection of studies implementing a sign-tracking protocol (lever CS). Rates of sign-tracking typically increase across training, whilst rates of goal-tracking initially increase then decline, eventually to below baseline levels. Error bars represent ±SEM

Fig. S2 Rates of magazine entry (goal-tracking) during the CS for groups trained on the sign-tracking cue. In saline-treated animals, goal-tracking behaviour was initially present, but then declined across training (as sign-tracking behaviour increased – see Figure 1). Treatment with α-flupenthixol impaired performance of goal-tracking on the sessions on which it was administered (1-7), and performance on subsequent drug-free sessions (8-12) showed the same pattern as was observed for the saline-treated animals at the beginning of training. Error bars represent ±SEM

Fig. S3 Rates of magazine entry during the PreCS period for groups trained on the goal-tracking procedure. Administration of α-flupenthixol impaired baseline rates of magazine responding (sessions 1-7), but this showed an immediate recovery in the post-treatment period (8-12). Error bars represent ±SEM

Fig. S4 Rates of magazine entry (goal-tracking) during the CS for groups trained on the sign-tracking cue. In saline-treated animals, goal-tracking behaviour was initially present, but then declined across training (as sign-tracking behaviour increased – see Figure 2). Treatment with SCH39166 impaired performance of goal-tracking on the sessions on which it was administered (1-7), and performance on subsequent drug-free sessions (8-12) showed the same pattern as was observed for the saline-treated animals at the beginning of training. Error bars represent ±SEM

Fig. S5 Panel **(a)** displays rates of magazine entry during the PreCS, while panel **(b)** displays rates of magazine entry (goal-tracking) during the CS, both for groups trained on the goal-tracking procedure. In the drug-treatment sessions (1-7), rates of magazine entry during the

PreCS and CS periods were impaired in the SCH39166-treated groups. In the post-treatment sessions (8-12), this impairment was no longer significant during the CS period, whereas in the PreCS period the effect was in the reverse direction; rates of magazine entry were significantly higher in the previously SCH39166-treated group. Error bars represent ±SEM

Fig. S6 Rates of magazine entry (goal-tracking) during the CS for groups trained on the sign-tracking cue. In saline-treated animals, goal-tracking behaviour was initially present, but then declined across training (as sign-tracking behaviour increased – see Figure 3). Treatment with eticlopride impaired performance of goal-tracking on the sessions on which it was administered (1-7), while performance on subsequent drug-free sessions (8-12) was higher in the previously eticlopride-treated animals than the saline-treated animals. Error bars represent ±SEM

Fig. S7 Panel **(a)** displays rates of magazine entry during the PreCS, while panel **(b)** displays rates of magazine entry (goal-tracking) during the CS, both for groups trained on the goal-tracking procedure. In the drug-treatment sessions (1-7), rates of magazine entry during the PreCS and CS periods were impaired in the eticlopride-treated groups. In contrast, rates of responding did not differ significantly between groups in either the PreCS or CS period across the post-treatment sessions (8-12). Error bars represent ±SEM

Fig. S1

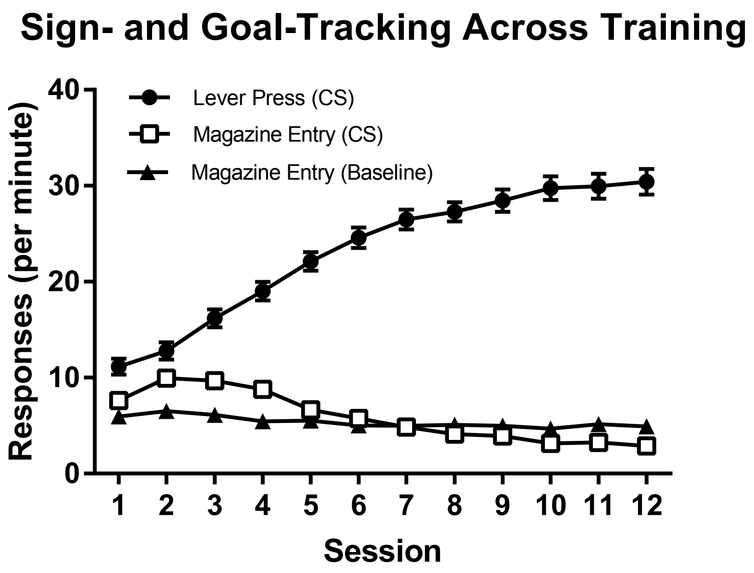
Sign- and Goal-Tracking Across Training
Lever Press (CS)
Magazine Entry (CS)
Magazine Entry (Baseline)
Responses (per minute)
0
10
20
30
40
1
2
3
4
5
6
7
8
9
10
11
12
Session

Fig. S2

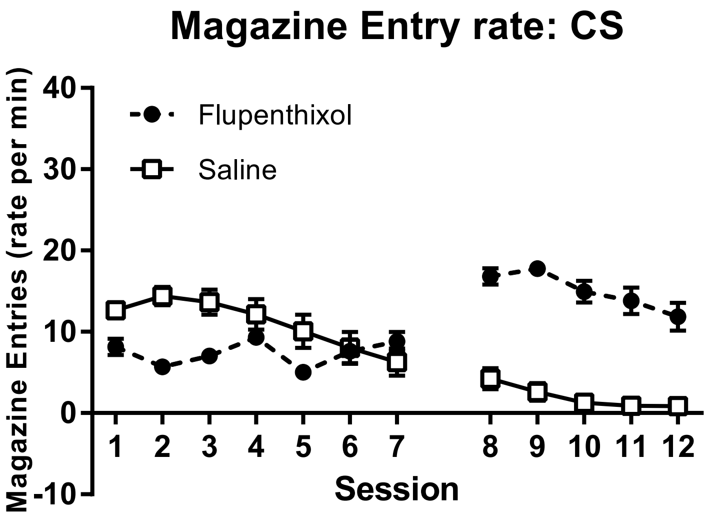

Magazine Entry rate: CS
Magazine Entries (rate per min)
40
30
20
10
0
-10
Flupenthixol
Saline
1
2
3
4
5
6
7
8
9
10
11
12
Session

Fig. S3

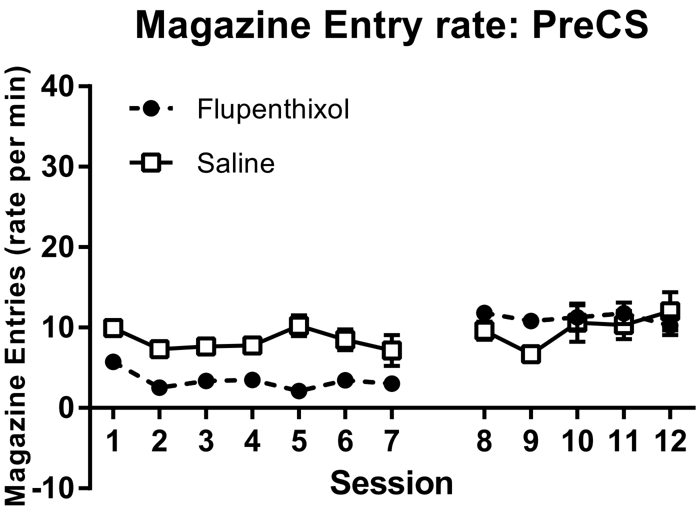
Magazine Entry rate: PreCS
Magazine Entries (rate per min)
40
30
20
10
0
-10
Flupenthixol
Saline
1 2 3 4 5 6 7 8 9 10 11 12
Session

Fig. S4

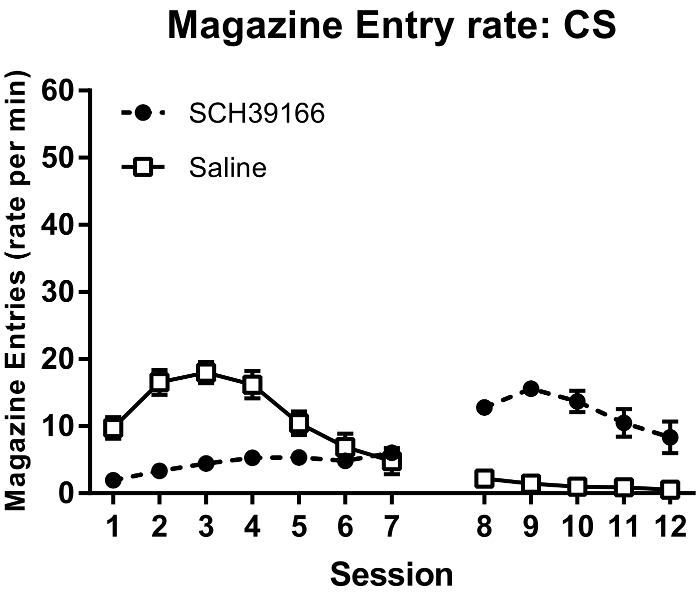
Magazine Entry rate: CS
SCH39166
Saline
Magazine Entries (rate per min)
60
50
40
30
20
10
0
1 2 3 4 5 6 7 8 9 10 11 12
Session

Fig. S5

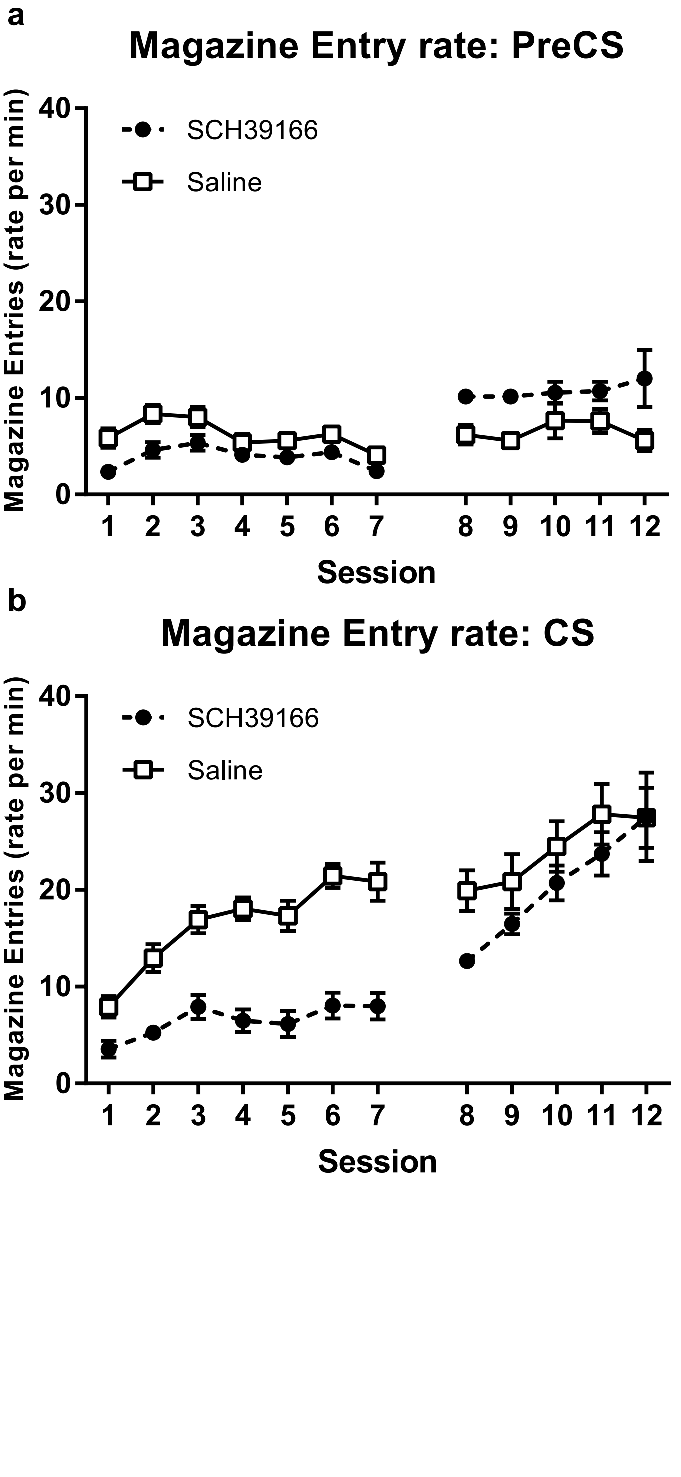
a
Magazine Entry rate: PreCS
Magazine Entries (rate per min)
SCH39166
Saline
0
10
20
30
40
1 2 3 4 5 6 7 8 9 10 11 12
Session
b
Magazine Entry rate: CS
Magazine Entries (rate per min)
SCH39166
Saline
0
10
20
30
40
1 2 3 4 5 6 7 8 9 10 11 12
Session

Fig. S6

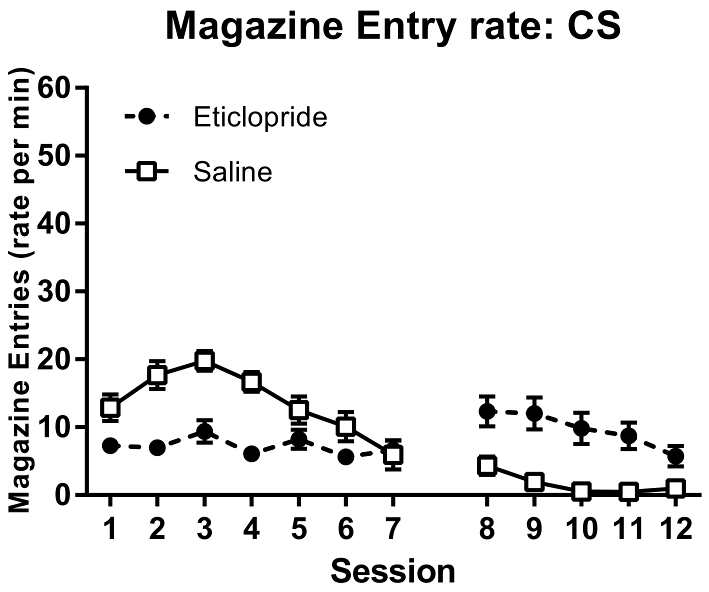

Magazine Entry rate: CS
Magazine Entries (rate per min)
60
50
40
30
20
10
0
Eticlopride
Saline
1
2
3
4
5
6
7
8
9
10
11
12
Session

Fig. S7

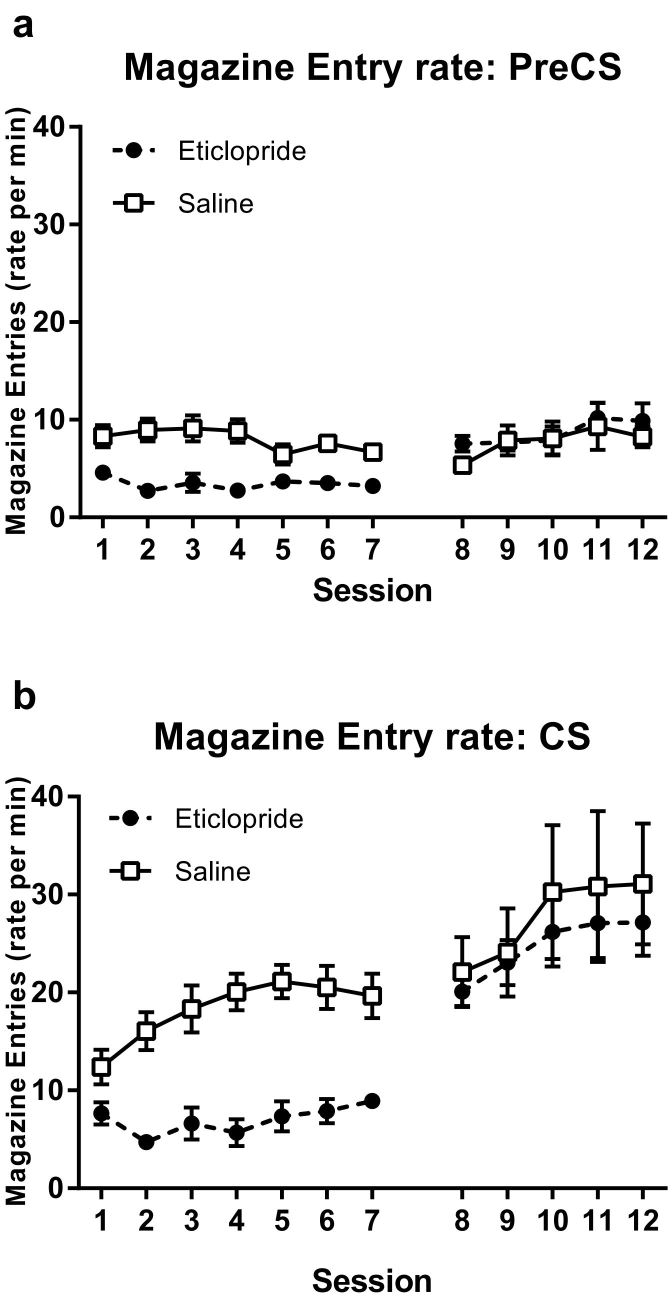
a
Magazine Entry rate: PreCS
Magazine Entries (rate per min)
Eticlopride
Saline
0
10
20
30
40
1 2 3 4 5 6 7 8 9 10 11 12
Session
b
Magazine Entry rate: CS
Magazine Entries (rate per min)
Eticlopride
Saline
0
10
20
30
40
1 2 3 4 5 6 7 8 9 10 11 12
Session
